# Proposed changes to the American Association of Physicists in Medicine governance

**DOI:** 10.1002/acm2.12124

**Published:** 2017-07-05

**Authors:** Todd Pawlicki, Rex G. Ayers, Kristy K. Brock, Jessica B. Clements, Bruce H. Curran, James T. Dobbins, Ehsan Samei, Eva Adams, Melissa Carol Martin, Lisa Schober

**Affiliations:** ^1^ TG281 UC San Diego; ^2^ TG281 Northwest Medical Physics Center; ^3^ TG281 UT MD Anderson Cancer Center; ^4^ TG281 Kaiser Permanente; ^5^ TG281 Virginia Commonwealth University Medical Center; ^6^ TG281 Duke University; ^7^ TG281 Duke University; ^8^ TG281; ^9^ TG281 Therapy Physics, Inc.; ^10^ TG281 AAPM

This issue's invited Editorial is provided by Todd Pawlicki, AAPM Secretary and Chair of Task Group No. 281, Governance Assessment Communications Plan. This is an opinion article and as such does not necessarily reflect the views and position of the JACMP EIC leadership team nor those of the JACMP Board of Editors. It is being provided to inform the medical physics community of the work and sentiments of TG‐281 and to assist the AAPM membership in its decision respecting the upcoming vote on this topic. –Michael Mills, JACMP EIC

The American Association of Physicists in Medicine (AAPM) was founded by physicists on November 17, 1958 in Chicago prior to the annual meeting of the Radiological Society of North America (RSNA). The AAPM was incorporated on November 10, 1965 with 16 members making up the Board of Directors (the Board). The Science, Professional, and Education Councils were created in 1972, 1973, and 1974, respectively. The Administrative Council was added later in 2010. Along the way, the AAPM has operated two highly successful journals, created many impactful technical documents, and has spearheaded other professional organizations such as the Commission on Accreditation of Medical Physics Education Programs (CAMPEP) and the Society of Directors of Academic Medical Physics Programs (SDAMPP). The membership has grown to over 8,500 and the Board has grown to 49 members (of which 38 are voting members). Over the past 59 yr, the AAPM has established itself as an internationally leading organization for the application of physics to medicine.

Given all of its successes and impact, the AAPM has not been without its refinements and course corrections. The American College of Medical Physics (ACMP) was started by the AAPM in the early 1980s only to be absorbed by AAPM in 2012. Likewise, the effort for licensure did not achieve its aim of requiring licensure for clinical medical physicists in all 50 States. The Annual Meeting has morphed over the years first to include a science track and later the education track followed by the professional track. Much later, the AAPM inherited the Spring Clinical Meeting from the ACMP partly because it better satisfied the practical needs of clinical physics members’ that were not being met by the annual meeting alone. So goes the life for an organization of dedicated and engaged members such as the AAPM; you win some, you lose some but are still able to maintain a positive impact on healthcare both at home and abroad.

It is prudent to describe the current governance structure of the AAPM. There are five officers of the AAPM—the President‐elect, President, Chair of the Board, Treasurer, and Secretary. This group along with the Executive Director constitutes the Executive Committee (EXCOM). EXCOM runs the day‐to‐day activities of the AAPM as well as performs Board‐related functions such as strategic decision making, fiduciary responsibilities, resource allocation, etc., in the intervals between Board meetings as provided by the AAPM Rules. There are 12 Board Members‐at‐large, 21 AAPM Chapter‐elected Board members, and 6 nonvoting members of the Board: Council Chairs, our Representative to the AIP Board, and the Executive Director. The President‐elect, Treasurer, Secretary, Members‐at‐large, and Chapter‐elected members are all elected by the General Membership or a subset of the General Membership (e.g., Chapter Membership). There are four Councils: Administrative, Education, Professional, and Science. The Chairs of these Councils are appointed positions. There is also a Strategic Planning Committee that is chaired by the Chair of the Board and has nine other appointed members.

With the complex healthcare landscape and new opportunities ahead, the AAPM has a responsibility to its members for continuous improvement. For the first time in many years, the AAPM has completed a critical assessment of itself and the ways in which it could do even better. About two and half years ago, the Ad Hoc Committee on Governance Assessment (AHCGA) was convened. This group, along with the Board of Directors and Strategic Planning Committee, has been assessing the current governance structure of the AAPM. The Board engaged a consulting group that specializes in nonprofit organizational governance, completed a membership survey (the first one in over 10 yr), and conducted several surveys of current AAPM leadership. As a result of this effort, several recommended changes to AAPM's governance structure are being proposed. These changes are tethered to the “DNA” of the organization and in concert with its ethos. For example, AAPM leadership should be made up of the various member stakeholders and constituencies with General Membership input through the elected positions. The purpose of this editorial is to describe the proposed changes in order to inform and inspire member readers as they cast their votes for this change.

Recognizing that the AAPM's core strength is its members, the AAPM must continue to be led by its major member stakeholder groups of clinical, science, chapters, educators, and members in both imaging and therapy subspecialties. As such, a new Council structure is being proposed to consist of the following Councils: Clinical Practice, Science, Regional Organization, Education, and Member Services.

The Clinical Practice Council will be focusing on stewardship of the profession of clinical medical physics with aspects of accreditation, credentialing, and practice guidelines constituting the purview of this Council. Communication with key collaborators will be an important task and include vendor relations, regulatory and legislative affairs, and patient communications as well as media response to clinical issues.

The Science Council will continue to function in much the same ways as it currently functions. It will have several responsibilities including: (a) oversee scientifically based policies and position papers, administer most of the scientific task groups and working groups through the Therapy Physics Committee and Imaging Physics Committee, (b) address research issues via the Research Committee, (c) provide assessment of technology through the Technology Assessment Committee, and (d) be responsible for press inquiries on scientific matters. A key aspect of its work will be to address the governmental aspects of research funding.

The Regional Organization Council is perhaps the most novel construct of the proposed structure and is being proposed in recognition that the profession has not realized optimal value from a collaboration of the AAPM with its Chapters. There are common needs of all chapters, regardless of geography, which can be addressed by identifying and focusing on them. Therefore, this Council will be tasked with ensuring synergy and coordination of Chapter activities with the broader AAPM efforts. Consequently, important areas to be addressed are education, vendor support of chapters, local regulatory issues, communication between the AAPM and Chapters as well as cultivation of future AAPM leaders. The Regional Organization Council will also work to identify Chapter needs and secure resources to help them achieve their specific goals.

The Education Council will maintain most of its current structure and charges but will now oversee both the International Educational Activities Committee and International Affairs Committee for better coordination of global outreach efforts.

The Member Services Council will carry important functions by focusing on the services and tools that will help members succeed in their profession. This Council will be responsible for publishing and the e‐presence of the AAPM including management of the association's journals. It will also handle AAPM meetings, awards and honors, and ensuring that AAPM's history is documented. Other important member needs will be addressed through the Membership Committee and Ethics Committee.

The proposal further recommends rightsizing the Board to 13 members consisting of the major member stakeholders. The new Board configuration calls for the following composition: the Chair of each of the Councils (5), the President‐elect, President, Chair of the Board, and Immediate Past Chair of the Board (4), the Treasurer (1), the Secretary (1), the Executive Director (1), and an optional non‐AAPM member (1). The purpose of allowing a nonmember to participate on the Board is to ensure expertise that is not typically obtainable through member appointments, e.g., hospital administrator, legal, marketing, or sales expert, or perhaps someone with experience in nonprofit revenue generation that does not increase the membership dues. It was felt that a Board seat would help get the most qualified person possible as well as establish a long‐term relationship for the benefit of AAPM and the profession rather than what one would get from a consultant. The non‐AAPM member Board position is not mandatory and will only be filled as the Board deems necessary.

As way of reference, two of our sister societies have similar governance structures as the newly proposed structure. On the imaging side, the RSNA with 54,000 members is a society comprised of radiologists, physicists, and other medical professionals that has an eight‐member Board that includes six Directors, the President, and the President‐elect. The Directors are liaisons for the cabinets of Education, Information Technology and the Annual Meeting, International Affairs, Publications and Communications, and Science. The President handles external relations and the President‐elect also serves as the Secretary‐Treasurer. On the therapy side, the American Society for Radiation Oncology (ASTRO) with over 10,000 members composed of physicians, nurses, biologists, physicists, therapists, dosimetrists, and other health care professionals is governed by a 15‐member Board. The Board consists of the Chair, President, President‐elect, Secretary‐Treasurer (one position), Immediate Past Chair, and the Chair and Vice‐chair for each of their Councils. Their five Councils are Clinical Affairs and Quality, Education, Government Relations, Health Policy, and Science.

The AAPM's governance proposal also includes the creation of a new operations group of 11 members — the Operations Committee — that will handle the day‐to‐day operations of the association, precluding the need for the AAPM's Executive Committee (EXCOM). The Council Vice‐chairs will make up the Operations Committee along with the President‐elect, President, Treasurer, Secretary, Government and Regulatory Affairs Committee Chair, and Executive Director (nonvoting).

The General Membership will elect future Board members as Vice‐chairs of the Councils, who will also first serve on the Operations Committee for 2 yr. Vice‐chairs will then succeed to Council Chairs and serve a two‐year term on the Board. The four‐member presidential chain (President‐elect, President, Chair of the Board, and Immediate Past Chair of the Board) will each serve one‐year terms. The Treasurer and the Secretary will serve up to two consecutive 2‐yr terms. Determination of candidates for the General Membership vote will be the responsibility of the Nominations Committee.

The Nominations Committee will have seven members. It will be chaired by the Immediate Past Chair of the Board. There will be one member from the Regional Organizations Council and four other members consisting of one person from academic therapy physics, one from community practice therapy physics, one from academic imaging physics, and one from community practice imaging physics. All will be staggered 2‐yr terms and be elected by a General Membership vote. In this way, the membership has input into the nominating process so that the AAPM leadership succession is open and transparent. The current Chair of the Board will be a nonvoting member. The purpose of the Nominations Committee is to make nominations for Council Vice‐chairs, President‐elect, Secretary, Treasurer, and the Nominations Committee.

Lastly, a Governance Committee, chaired by the Secretary, will be created and tasked with several key functions not currently performed within the AAPM but crucial to good organizational governance. It will be tasked with routinely and critically assessing the effectiveness of the AAPM's governance, ensuring diversity of Board members, as well as developing and ensuring the necessary knowledge and governance skills of Board members.

In closing, it is important to remember that when making a change like this, “perfect” is the enemy of “better”. Not every issue will be automatically addressed with the proposed changes and not every change will suddenly produce fantastic results. Over the years however, one constant for the AAPM has been change. In keeping with that tradition, the above‐described governance changes are just the next step in a natural evolution of the AAPM. The proposed changes will be presented for a General Membership vote shortly after the Annual Meeting in Denver. TG‐281 encourages you to cast your vote on August 23, 2017 and play a vital role in shaping the future of your organization.

AAPM Task Group No. 281 Governance Assessment Communications Plan:

